# Lower dietary phosphorus supply in pigs match both animal welfare aspects and resource efficiency

**DOI:** 10.1007/s13280-017-0969-8

**Published:** 2017-11-20

**Authors:** Michael Oster, Christian Gerlinger, Kaja Heide, Franziska Just, Luisa Borgelt, Petra Wolf, Christian Polley, Brigitte Vollmar, Eduard Muráni, Siriluck Ponsuksili, Klaus Wimmers

**Affiliations:** 1 Institute for Genome Biology, Leibniz Institute for Farm Animal Biology (FBN), Wilhelm-Stahl-Allee 2, 18196 Dummerstorf, Germany; 2 University of Rostock, Justus-von-Liebig-Weg 6b, 18059 Rostock, Germany; 3 LUFA-ITL GmbH, Dr.-Hell-Str. 6, 24107 Kiel, Germany; 4 Institute for Experimental Surgery, University Medical Center Rostock, Schillingallee 69a, 18057 Rostock, Germany; 5 Faculty of Agricultural and Environmental Sciences, University of Rostock, Justus-von-Liebig-Weg 6b, 18059 Rostock, Germany

**Keywords:** Calcium–phosphorus ratio, Gene expression, Growth, Phosphorus efficiency, Pig, Vitamin D

## Abstract

Dietary phosphorus frequently exceeds age-specific requirements and pig manure often contains high phosphorus load which causes environmental burden at regional scales. Therefore, feeding strategies towards improved phosphorus efficiency and reduced environmental phosphorus load have to be developed. A 5-week feeding trial was conducted: piglets received medium, lower (−25%), or higher (+25%) amounts of phosphorus and calcium. Dietary responses were reflected by performance parameters, bone characteristics, and molecular data retrieved from serum, intestinal mucosa, and kidney cortex (*p* < 0.05). Transcripts associated with vitamin D hydroxylation (*Cyp24A1*, *Cyp27A1*, *Cyp27B1*) were regulated by diet at local tissue sites. Low-fed animals showed attempts to maintain mineral homoeostasis via intrinsic mechanisms, whereas the high-fed animals adapted at the expense of growth and development. Results suggest that a diet containing low phosphorus and calcium levels might be useful to improve resource efficiency and to reduce phosphorus losses along the agricultural value chain.

**Electronic supplementary material:**

The online version of this article (10.1007/s13280-017-0969-8) contains supplementary material, which is available to authorized users.

## Introduction

The sufficient availability of high-quality agricultural products relies on the sustainable usage of resources. Phosphorus (P) is an irreplaceable component of life and, thus, widely used in all agricultural production systems. However, in pig husbandry the dietary P levels often exceed animal- and age-specific requirements. Pigs are considered to be major excretors of P from agricultural systems which lead to a severe environmental burden. In order to balance economic and environmental sustainability related to the uneven density of animal production, novel approaches of P management aim to slow down the rate of emissions through transformative or incremental system-wide processes (Kebreab 2013). This includes knowledge and measures to reduce P excretion targeting benefits for animal health and environment.

**Fig. 1:**
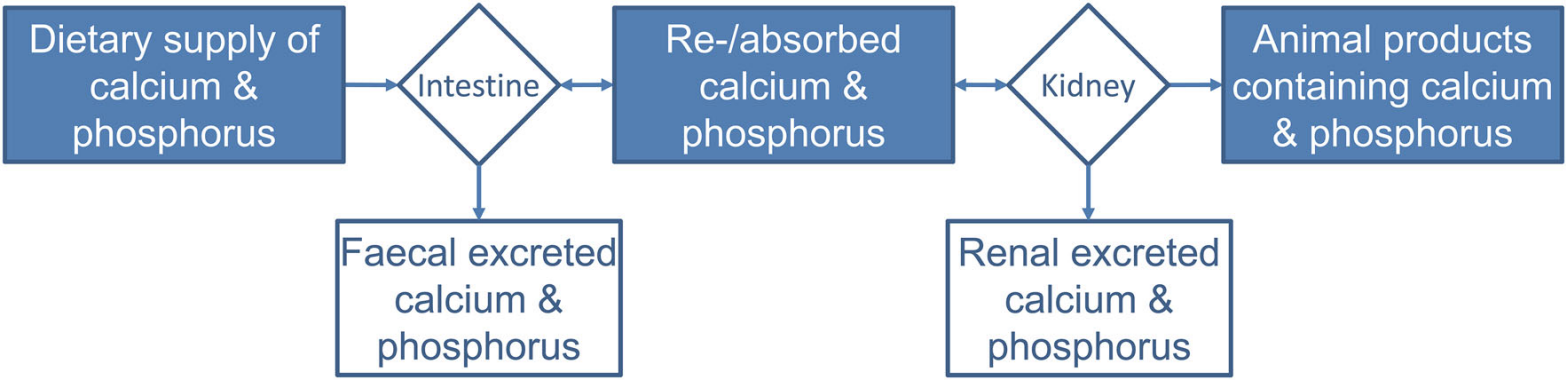
The flow of calcium and P from diet to animal products and slurry

Decreased intake of P by farm animals results in lowered P excretion but also lowered P retention. At high P supply, P absorption and excretion will continue to increase, while P retention will reach a plateau at certain amounts (Rodehutscord et al. 1999). Hence, an optimal P efficiency requires a high P absorption, a sufficient skeletal storage, and a low P excretion (Fig. 1) (Poulsen 1994). The addressed mechanisms are interlinked with dietary calcium levels (Berndt and Kumar 2009; Taylor and Bushinsky 2009).

Feeding regimens containing variable calcium and P contents are known to affect related endocrine levels in mammals (Proszkowiec-Weglarz and Angel 2013; Oster et al. 2016), such as vitamin D (calcitriol) and parathyroid hormone (PTH). The serum levels of both hormones are interdependent due to sophisticated feedback mechanisms involving receptors and transporters localised in the small intestine, bone, and kidneys (Berndt and Kumar 2009). Hence, organismal requirements are ensured by affecting re-/absorbing and excreting tissues. Since PTH regulates the activation of calcitriol via 1α-hydroxylase (Friedman et al. 1996), a P-deficient diet was associated with increased renal mRNA abundances of the 1α-hydroxylase (*Cyp27B1*) (Alexander et al. 2008). Consequently, both activity and intracellular redistribution of sodium-dependent phosphate transporters (SLC34 family) rely on dietary P levels in porcine jejunum (Saddoris et al. 2010) and murine kidney cells (Lanaspa et al. 2013). Moreover, the mineralisation of the skeleton relies on dietary P supplies as indicated by trabecular bone characteristics such as bone mineral density and structure model index (Oster et al. 2016). Interestingly, recent studies suggested that both deficient and excessive P supplies may have negative effects on bone mineralisation and health (Sørensen 2012).

The characterisation of physiological and molecular processes enabling increased P efficiency in monogastric species is crucial towards a P-resilient livestock production. The study investigates the effects of wheat/barley-based diets varying in calcium and P contents on growing pigs. Six tissues and body compartments including serum, bone, duodenum, jejunum, colon, and kidney cortex were used to detect mechanisms of adaptation. We monitored a number of traits, including (i) performance and growth data, (ii) calcium and P mineral homoeostasis, (iii) bone characteristics, and (iv) expression of candidate genes which represent the receptors of relevant hormones, their in-/activating enzymes and P transporters.

## Materials and methods

### Animals and diets

The study was approved by the Scientific Committee of the FBN, and the experimental setup was generally licensed by the ethics committee of the federal state of Mecklenburg-Western Pomerania, Germany (Landesamt für Landwirtschaft, Lebensmittelsicherheit und Fischerei; LALLF M-V/TSD/7221.3-1-053-15). Twenty-one German Landrace piglets obtained from four litters were randomly assigned to one of three wheat/barley/soybean-based diets (Table S1). Offspring were sired by four boars. The study comprised three to four piglets per sex and dietary group. From weaning (day 28) until slaughter (day 64), piglets received a diet containing low (L, calcium: 0.79%; P: 0.56%), medium (M; calcium: 1.27%; P: 0.84%), or high calcium and P levels (H; calcium: 1.69%; P: 1.02%). The dietary P content in compound feed (group M) corresponded to current recommendations (GfE 2006). Dietary compositions resulted in only slight variation of the calcium:P-ratios (L: 1.41; M: 1.51; H: 1.65). The achieved dietary levels of digestible P were 0.31% (L), 0.49% (M), and 0.60% (H). Neither phytase nor other phosphatases were added. Piglets were individually reared in cages on flat decks in environmentally controlled rooms. The animals had ad libitum access to pelleted feed and water. Piglets were weighed weekly. Feed intake was quantified and feed conversion ratio (FCR) was calculated.

### Collection and preparation of serum samples

At day 64, pigs were narcotised by electrical stunning and sacrificed by exsanguination in the experimental slaughterhouse of FBN. Blood samples were collected from trunk blood. Serum was prepared and samples were stored at −80 °C until use. Tissue sampling comprised intestinal mucosa and kidney cortex which were immediately collected, frozen in liquid nitrogen, and stored at −80 °C. Intestinal samples of ~5 cm in length have been taken from the duodenum (10 cm distal from the pyloric junction), jejunum (1 m distal from the pyloric junction), and the anterior part of the colon (Patterson et al. 2008). Intestinal sections have been washed with PBS to ensuring removal of all feed residues.

### Serum hormones and mineral measurements

Serum calcitriol (Immunodiagnostic Systems, Frankfurt am Main, Germany) and PTH (Immundiagnostik, Bensheim, Germany) were determined in duplicate using commercially available enzyme-linked immunosorbent assays (ELISA) according to manufacturer’s protocols. Moreover, serum cortisol and serum thyroid hormones triiodothyronine (T_3_) and thyroxin (T_4_) were determined in duplicate using a commercially available magnetic bead-based quantitative immunoassay (MAGPIX system) according to manufacturer’s protocols (Merck Millipore, Darmstadt, Germany). Mineral serum measurements (inorganic P, calcium) were analysed with commercial assays using Fuji DriChem 4000i (FujiFilm, Minato, Japan).

### Chemical analyses of bones

The individual left femurs were collected at slaughter (day 64) and stored at −20 °C until use. The proximal part (30% of total length) of the bones was separated and lyophilised. Subsequently the bones were degreased. The bone tissue was comminuted by ball mill after embrittlement in liquid nitrogen. Wet chemical analyses according to common methods of VDLUFA were applied (Naumann et al. 1976). Contents of crude ash as well as calcium and P were determined.

### RNA isolation

Total RNA was isolated using TRI Reagent per manufacturer’s directions (Sigma-Aldrich, Taufkirchen, Germany), then treated with DNase and purified with the column-based NucleoSpin RNA II-Kit (Macherey–Nagel, Düren, Germany). RNA integrity was determined by visualisation on a 1% agarose gel containing ethidium bromide and the concentration was measured using the NanoDrop ND-1000 spectrometer (PEQLAB, Erlangen, Germany). Absence of DNA contamination was verified by PCR amplification of the porcine *RPL32* gene (forward primer: 5′-AGCCCAAGATCGTCAAAAAG-3′; reverse primer: 5′-TGTTGCTCCCATAACCAATG-3′). All RNA samples were stored at −80 °C. First-strand cDNA was synthesised from 2 μg of total RNA using random primers (Promega, Fitchburg, WI, USA) and oligo d(T) 13VN in the presence of Superscript III reverse transcriptase (Invitrogen, Karlsruhe, Germany). The final cDNA was diluted with Aqua dest to a total volume of 100 µl.

### Quantitative real-time PCR (qRT-PCR)

On the transcriptional level, receptors of calcitriol, PTH, and thyroid hormones were analysed, i.e. *VDR* (vitamin D receptor), *PTH1R* (parathyroid hormone 1 receptor), and *THRA* (thyroid hormone receptor alpha). Furthermore, genes encoding calcitriol in-/activating enzymes were analysed, i.e. *Cyp24A1* (vitamin D_3_ 24-hydroxylase), *Cyp27A1* (vitamin D_3_ 25-hydroxylase), and *Cyp27B1* (1α-hydroxylase). Moreover, analyses of P transporters comprise *SLC34A1* (solute carrier family 34, member 1; NaPi2a), *SLC34A2* (solute carrier family 34, member 2; NaPi2b), and *SLC34A3* (solute carrier family 34, member 2; NaPi2c).

Transcript levels of selected target (*VDR*, *Cyp24A1*, *Cyp27A1*, *Cyp27B1*, *PTH1R*, *THRA*, *SLC34A1*, *SLC34A2*, *SLC34A3*) and reference genes (*RPL32*) were quantified by qRT-PCR. Individual mRNA samples (*n* = 21 per tissue) were analysed in duplicate on a LightCycler 480 system using the LightCycler 480 SYBR Green I Master (Roche, Mannheim, Germany) according to manufacturer’s instructions. Briefly, reactions were performed in a final volume of 10 µl using 5.0 µl of LightCycler 480 SYBR Green I Master (Roche), 0.5 µl (10 µM) of each primer (Table S2), 2 µl (40 ng) cDNA, and 2.0 µl of Aqua dest. The temperature profiles comprised an initial denaturation step at 95 °C for 10 min followed by 40 cycles consisting of denaturation at 95 °C for 15 s, annealing at 60 °C for 10 s, and extension/fluorescence acquisition at 72 °C for 15 s. Amplified products were subjected to melting curve analyses and gel electrophoresis to verify the absence of non-specific products. For all the assays, threshold cycles were converted to copy numbers using a standard curve generated by amplifying serial dilutions of a corresponding PCR standard (10^7^–10^1^ copies). Transcripts with a mean ≤5 copies per 10 ng RNA transcribed were considered as not detectable (nd).

### Data analyses

Data referring to gene expression, physiological traits, hormones, and bone measurements were analysed via variance analyses (PROC MIXED; SAS version 9.4; SAS Institute, Cary, NC, USA), including effects represented by dietary group, sex, and sire (*V*_ijk_ = µ + diet_i_ + sex_j_ + sire_k_ + error_ijk_). The retrieved LSmeans were compared using Tukey’s post hoc test. The level of significance was set at *p* < 0.05.

## Results

### Piglet performance and feed conversion ratio

High levels of dietary calcium and digestible P revealed decreased live weights compared to low- and medium-fed animals (Table 1). Moreover, cumulative daily feed intake and daily body weight gain were decreased in high-fed animals which resulted in an increased feed conversion ratio (FCR).

**Table 1:** Performance traits of pigs fed experimental diets with low, medium, and high calcium and digestible P contents

Item	Unit	Low		Medium		High	
		LSmean	SE	LSmean	SE	LSmean	SE
Live weight (day 28)	kg	8.4	0.4	8.4	0.4	8.4	0.4
Live weight (day 64)	kg	23.2^b^	1.2	20.8^b^	1.2	15.7^a^	1.2
Daily feed intake (day 28–day 64)	g/day	691^b^	28	622^a,b^	28	549^a^	28
Daily body weight gain (day 28–day 64)	g/day	403^b^	29	368^b^	29	198^a^	29
FCR (day 28–day 64)	g/g	1.6^a^	0.5	1.7^a^	0.5	3.4^b^	0.5

^a,b^Indicate significant differences between groups (*p* < 0.05)

### Diet-specific serum hormone levels and mineral measurements

As displayed in Table 2, serum calcitriol was increased in L animals but decreased in H animals (L > M > H). Regarding parathyroid hormone (PTH), dietary effects were observed between L and H samples (L < H). T_3_ was decreased in H animals (L > H; M > H). No significant dietary effects on serum T_4_ levels, cortisol, inorganic P, and calcium were observed.

**Table 2:** Serum measurements of pigs fed experimental diets with low, medium, and high calcium and digestible P contents

Item	Unit	Low		Medium		High	
		LSmean	SE	LSmean	SE	LSmean	SE
Calcitriol	pmol/l	572.68^c^	26.61	392.42^b^	37.07	291.95^a^	26.61
Parathyroid hormone	pg/ml	2.26^a^	2.46	6.19^a,b^	2.71	10.51^b^	2.30
T_3_	ng/ml	0.81^b^	0.08	0.82^b^	0.08	0.47^a^	0.08
T_4_	ng/ml	11.64	1.24	10.62	1.24	11.90	1.24
Cortisol	ng/ml	114.76	11.52	122.45	11.48	123.98	11.52
Inorganic phosphorus	mg/dl	10.02	0.50	10.45	0.49	10.61	0.50
Calcium	mg/dl	9.61	0.29	9.64	0.29	9.51	0.29

^a,b,c^Indicate significant differences between groups (*p* < 0.05)

### Bone characteristics

Femur length was unaffected by diet (Table 3). The fat-free dry matter of the femur (DM_fat free_) was decreased in L animals compared to M and H animals (L < M; L < H). Regarding crude ash, dietary effects were observed between L and H samples (L < H). No significant differences were observed for femoral calcium and P measures and calculated femoral calcium–P ratio.

**Table 3:** Femur characteristics of pigs fed experimental diets with low, medium, and high calcium and digestible P contents

Item	Unit	Low		Medium		High	
		LSmean	SE	LSmean	SE	LSmean	SE
Femur length	cm	12.7	0.6	12.8	0.7	12.2	0.5
DM_fat free_	mg/g FM	296^a^	19.6	345^b^	16.7	345^b^	14.0
Crude ash	mg/g DM_fat free_	417^a^	52.7	476^a,b^	51.5	487^b^	53.3
Calcium	mg/g DM_fat free_	171	14.8	208	53.9	210	36.7
Phosphorus	mg/g DM_fat free_	73.4	10.4	82.2	12.1	92.2	30.4
Calcium:phosphorus ratio		2.4	0.4	2.6	0.9	2.5	1.0

^a,b^Indicate significant differences between groups (*p* < 0.05)

### Gene expression in re-/absorbing and excreting tissues

The dietary challenges revealed site-specific transcriptional responses in duodenum, jejunum, colon, and kidney (Table 4). In duodenum, mRNA abundances of *Cyp24A1* (L > M; L > H) and *SLC34A3* differed significantly between dietary groups (L < H). In jejunum, genes encoding for *Cyp24A1* (L > M; L > H; M > H), *Cyp27B1* (L < H), *THRA* (M < H), and *SLC34A3* (L > H) were diet-dependently altered. In colon, *THRA* (L < H) and *Cyp27B1* were higher expressed in H animals (L < H; M < H). Gene expression in kidney cortex revealed lower mRNA abundances of *VDR* in L animals (L < M; L < H). Renal expression of *THRA* was increased in H animals (L < H; M < H), whereas genes encoding *Cyp27A1* and *Cyp27B1* were higher expressed in L animals (L > M; L > H). Moreover, mRNA abundances of *SLC34A3* differed between H and L animals (L > H). Genes encoding for *SLC34A1* and *SLC34A2* were not detectable in duodenum, jejunum, and colon, whereas *SLC34A3* was expressed in all analysed tissues.

**Table 4:** Tissue-specific relative gene expression and copy numbers of selected transcripts in pigs fed experimental diets with low, medium, and high calcium and digestible P contents. Significant differences are displayed in bold. *FC* fold change, *nd* not detectable

Tissue	Gene symbol	Low versus medium FC	*p*	Low versus high FC	*p*	Medium versus high FC	*p*	Copy number^a^		
								Min	Max	Mean
Duodenum	VDR	+ 1.47	0.148	**−** 1.17	0.579	**− **1.73	0.065	1599	4327	2709
	Cyp24A1	**− 5.69**	**0.049**	**− 4.09**	**0.044**	+ 1.39	0.858	6	225	45
	CYP27A1	+ 1.06	0.884	+ 1.31	0.396	+ 1.24	0.514	2859	15 202	6949
	CYP27B1	+ 1.26	0.272	+ 1.05	0.820	**−** 1.20	0.379	8	31	16
	PTH1R	+ 1.12	0.625	+ 1.03	0.904	**−** 1.09	0.706	16	92	47
	THRA	+ 1.13	0.583	+ 1.16	0.458	+ 1.03	0.870	317	1098	598
	SLC34A1	nd	nd	nd	nd	nd	nd	nd	nd	nd
	SLC34A2	nd	nd	nd	nd	nd	nd	nd	nd	nd
	SLC34A3	**−** 1.13	0.662	**− 2.47**	**0.032**	**−** 2.18	0.095	84	762	268
Jejunum	VDR	+ 1.05	0.777	**− **1.38	0.149	**− **1.45	0.092	823	4604	2206
	Cyp24A1	**− 45.37**	**< 0.001**	**− 528.81**	**< 0.001**	**− 11.66**	**0.006**	0	412	65
	CYP27A1	**−** 1.52	0.239	**−** 1.43	0.299	+ 1.07	0.876	35	769	313
	CYP27B1	+ 1.25	0.439	**+ 1.86**	**0.015**	+ 1.49	0.072	3	15	8
	PTH1R	**−** 1.27	0.558	+ 1.45	0.217	+ 1.83	0.081	2	20	9
	THRA	**−** 1.18	0.245	+ 1.23	0.092	**+ 1.45**	**0.009**	728	1843	1169
	SLC34A1	nd	nd	nd	nd	nd	nd	nd	nd	nd
	SLC34A2	nd	nd	nd	nd	nd	nd	nd	nd	nd
	SLC34A3	**−** 1.36	0.130	**− 1.86**	**0.013**	**− **1.37	0.246	485	2698	1612
Colon	VDR	+ 1.08	0.673	**− **1.17	0.449	**− **1.26	0.248	722	2874	1602
	Cyp24A1	nd	nd	nd	nd	nd	nd	nd	nd	nd
	CYP27A1	**− **1.23	0.348	**− **1.32	0.213	**− **1.08	0.750	110	543	262
	CYP27B1	+ 1.94	0.077	**+ 3.33**	**< 0.001**	**+ 1.72**	**0.014**	12	74	34
	PTH1R	+ 1.27	0.368	+ 1.49	0.116	+ 1.17	0.474	24	124	52
	THRA	+ 1.21	0.155	**+ 1.45**	**0.006**	+ 1.20	0.111	533	1236	910
	SLC34A1	nd	nd	nd	nd	nd	nd	nd	nd	nd
	SLC34A2	nd	nd	nd	nd	nd	nd	nd	nd	nd
	SLC34A3	+ 1.29	0.242	+ 1.36	0.145	+ 1.06	0.761	10	33	18
Kidney	VDR	**+1.93**	**0.001**	**+1.99**	**< 0.001**	+ 1.03	0.781	547	2175	1240
	Cyp24A1	+ 3.82	0.072	+ 1.74	0.613	**−** 2.19	0.175	17	5723	1248
	CYP27A1	**− 1.57**	**0.006**	**− 1.61**	**0.005**	**− **1.02	0.913	467	2256	1194
	CYP27B1	**− 2.01**	**<0.001**	**− 1.93**	**< 0.001**	+ 1.05	0.803	517	3097	1274
	PTH1R	+ 1.02	0.808	+ 1.02	0.805	+ 1.01	0.998	5379	10 662	8235
	THRA	+ 1.07	0.444	**+ 1.28**	**0.010**	**+ 1.19**	**0.048**	957	1881	1265
	SLC34A1	+ 1.02	0.854	**− **1.13	0.205	**− **1.15	0.154	35 633	67 928	53 221
	SLC34A2	**−** 1.45	0.161	**−** 1.05	0.814	+ 1.38	0.234	3	19	9
	SLC34A3	**− **1.12	0.154	**− 1.22**	**0.020**	**− **1.09	0.286	696	1363	1054

^a^mRNA copies per 10 ng RNA transcribed

### Trait relationships

**Fig. 2:**
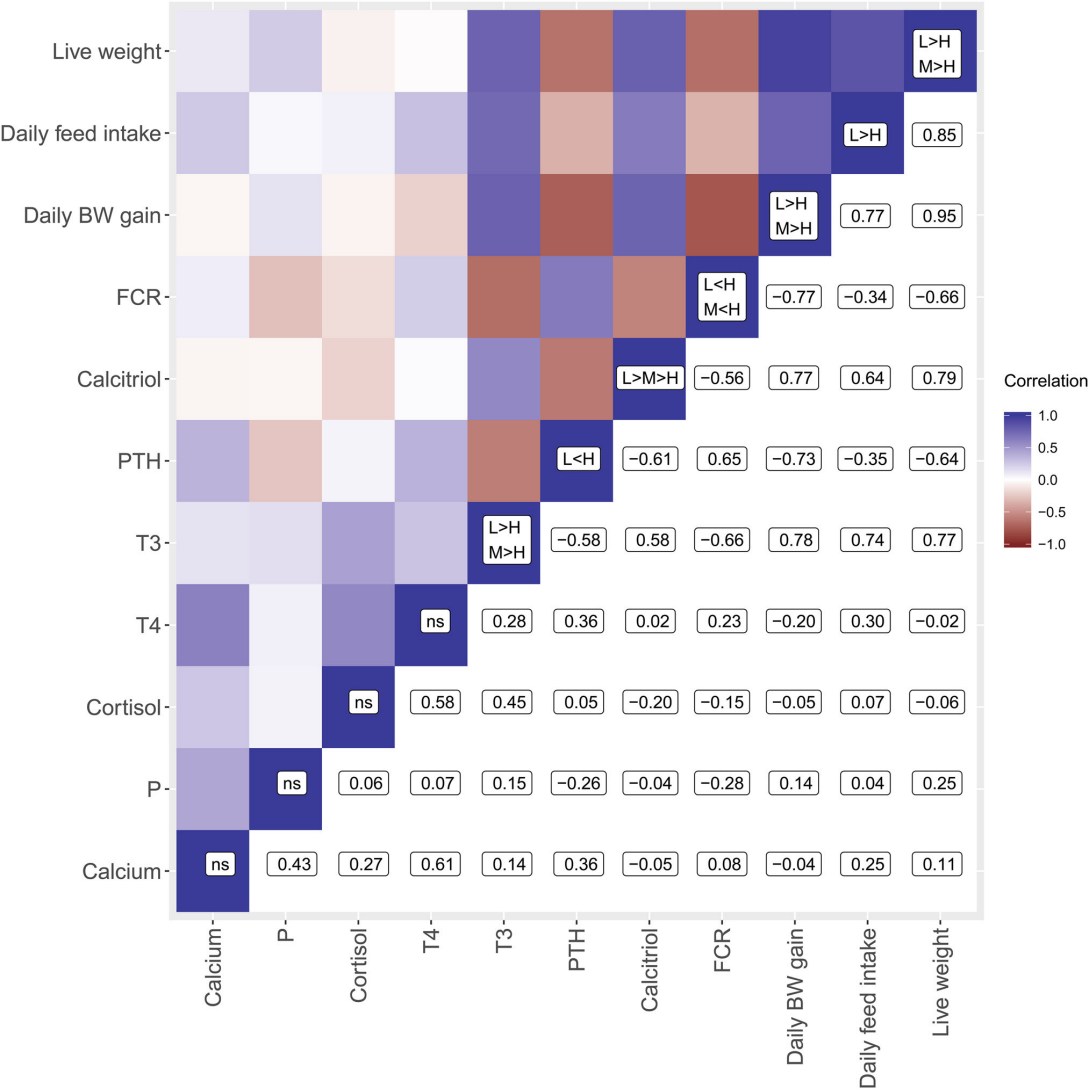
Heatmap displaying Pearson correlation coefficients comprise performance traits and serum measurements. The diagonals summarise significant alterations indicated in Tables 1 and 2

PTH showed negative linear correlations with calcitriol and T_3_, whereas the latter ones were positively correlated (Fig. 2). Cortisol and serum calcium levels showed positive linear correlations with T_4_. Serum P was not correlated to any tested trait. Live weight, daily feed intake, and daily BW gain were positively correlated with calcitriol and T_3_ but negatively correlated with PTH.

## Discussion

Reaching sustainability will require tracking the fate of P and to understand how we can decrease P losses from the agri-environment system and tighten the resource cycle. Indeed, diet and animals are considered the most important determining factors driving P efficiency. The P demand should match genetic and physiological requirements to avoid P excess in manure. In this context, the broad biodiversity of monogastric P utilisation has been described (Hittmeier et al. 2006; Alexander et al. 2008).

The higher calcium and P supply in H animals prompted a lower feed intake, lower body weight gain, and higher FCR. Consequently, the study pointed out molecular routes that are responsive to such dietary challenges and, therefore, represented molecules and genes related to P efficiency. In this study, calcitriol and PTH levels but not serum calcium and P levels were affected by diet. Indeed, endocrine factors such as calcitriol and PTH are known to coordinate serum calcium and P levels (Dusso et al. 2005; Talmage and Mobley 2008). Indeed, calcitriol and PTH were negatively correlated (Fig. 2) to balance enteral absorption, osseous mobilisation, and renal excretion rates as previously reviewed (Berndt and Kumar 2009). Obviously, the endocrine response to diets varying in calcium and P levels enabled serum mineral homoeostasis. According to previous studies (Engstrom et al. 1985; Sommerville et al. 1985; Riond et al. 2001; Oster et al. 2016), lowered PTH levels but increased calcitriol levels reflect the organismal effort to minimise urinary calcium and P losses and to enhance enteral calcium and P absorption in L samples. In contrast, H samples aimed to maximise renal calcium and P losses via lower calcitriol and higher PTH levels.

To regulate mineral homeostasis within the intra- and extracellular fluid, maturation and development provoke dynamic influxes and effluxes of calcium and P which is largely buffered by the osseous storage. Calcitriol and PTH also have an impact on bone tissue via control of diet-specific osteoblast- and osteoclast-mediated actions. It became obvious that calcitriol and PTH responses were sufficient to maintain physiological calcium and P serum concentrations by recruiting the bone mineral storage. Hence, results suggest an altered bone mineralisation when comparing L and H animals which is in agreement with previous studies (Ryan et al. 2011; Varley et al. 2011). Moreover, the skeleton is an important target tissue of thyroid hormones such as T_3_, which controls bone turn-over and maintenance throughout life (Williams 2013; Bassett and Williams 2016). In our study, the reduced T_3_ levels in H animals compared to L animals reflect the observed decline in feed intake (Wadden et al. 1990) and may account for impaired bone resorption and formation phases via reduced osteoblast differentiation and function. In fact, reduced T_3_ levels have been associated with increased bone mineralisation and higher risk of fracture (Vestergaard et al. 2005; Tuchendler and Bolanowski 2014). Interestingly, the decreased endocrine T_3_ levels observed in serum of H animals were accompanied by increased abundances of *THRA* encoding the thyroid receptor alpha. Since T_3_ action relies on both hormone availability and mRNA copy number, results might suggest adaptation processes at the receptor level (Kenessey and Ojamaa 2004) to match, e.g. intestinal demands modulating cell proliferation rates (Plateroti et al. 2006).

However, since T_3_ impacts on growth via anabolic and catabolic processes, the reduced T_3_ levels reflect the lower body weight and BW gain as shown by correlation analyses (Fig. 2). It will be of great scientific interest to find out which molecular mechanisms are able to sense the high calcium and P content in the diet which lead to a lower feed intake but increased FCR. Indeed, it may be conceivable that differences for feed intake might have affected endocrine responses such as for T_3_. Interestingly, the low P supply did not affect growth and feed efficiency; it even tended to improve performance, while mineral homeostasis was maintained. The animals were able to cope with lowered P supply at least over the period of time tested here.

The endocrine responses to both L and H diets indicate the interplay between intestine, bone, and kidney. Whereas mRNA abundances of *PTH1R* were unaltered in all analysed tissues, the higher abundances of *Cyp27A1*, and *Cyp27B1* in kidney mirrored the increased calcitriol levels on L diet. These results are in accordance to previous reports when P-restricted diets have been applied to mice (Zhang et al. 2002). Additionally, *Cyp27B1* expression was affected in jejunum and colon tissues, although the mRNA copy number was rather low. However, the tissue-specific increased abundances of *Cyp27B1* in H animals might account for a local calcitriol synthesis in the intestine required for, e.g. immunological aspects (Dusso et al. 2005; Liu et al. 2006). In this context, local requirements for calcitriol might be balanced via significantly different *Cyp24A1* expression in duodenum and jejunum as the encoded 24-hydroxylase catalyses the first step in the deactivation of calcitriol (Sakaki et al. 2005). Indeed, the dietary challenge in this study revealed a strong transcriptional response of *Cyp24A1* at local tissue sites. Moreover, *VDR* was diet-specifically expressed in kidney but not in intestine. The higher renal abundances of VDR might reflect a compensatory regulation of L animals to achieve a mineral balance. However, VDR is known to initiate various effects in different tissues including intestine and kidney. Specifically, effects were mediated via the VDR–RXR receptor heterodimer binding to vitamin D response elements which are detected in the promoter region of a broad range of genes (Haussler et al. 2013). Despite unaffected *VDR* in intestine tissues, however, it is conceivable that dietary effects are mediated since calcitriol serum levels were very high in L animals.

Regarding the P transporter represented by the SLC34 family, intestinal *SLC34A1* and *SLC34A2* were at the lower detection limit, whereas renal *SLC34A1* was highly abundant. In contrast, *SLC34A2* was highlighted as relevant P transporter in the posterior parts of the small intestine in mice (Radanovic et al. 2005). In particular, it has been proposed that *SLC34A2* is responsible for transcellular P uptake in jejunum and its mRNA expression is upregulated by decreased P levels in chickens (Li et al. 2012) and rats (Cao et al. 2016). Obviously, this does not reflect the porcine responses revealed in this study. In our study, H animals showed lower abundances of *SLC34A3* in duodenum and jejunum but higher abundances in kidney when compared with L animals. This pattern might follow superior endocrine responses to minimise the P influx via intestine and primary urine. Correspondingly, it has been shown that *SLC34A3* is specifically regulated in response to high P diets in rats (Segawa et al. 2005). However, it has been proposed that mechanisms involved in P transport worked independently of transcriptional events (Saddoris et al. 2010).

## Conclusion

The responses to the diet containing low calcium and P levels were sufficient to maintain physiological calcium and P serum concentrations by recruiting the bone mineral storage. However, the diet containing high calcium and P levels revealed to be inappropriate for an adequate growth performance since a negative effect of high dietary calcium–P levels on feed intake was observed. Indeed, the dynamic influxes and effluxes of calcium and P among organs and tissues were reflected by the pronounced endocrine and transcriptional responses and might be of critical importance to produce P-resilient phenotypes. The intestinal mucosa and kidney cortex were highlighted as initial sites to maintain mineral homoeostasis. The observed responses contribute to implement feeding strategies to preserve global P resources and to reduce agricultural residues. Results clearly suggest that the usage of calcium and P should be monitored and better regulated within a framework of improved governance. Current feeding recommendations for livestock systems need to consider aspects for animal health as well as economic and environmental perspectives to reduce dietary mineral intake in growing pigs.

## Electronic supplementary material

Below is the link to the electronic supplementary material.

Supplementary material 1 (PDF 194 kb)
