# Supplementary material for: Lower dietary phosphorus supply in pigs match both animal welfare aspects and resource efficiency
Source: Ambio. 2017 Nov 20;47(Suppl 1):20–9. doi: 10.1007/s13280-017-0969-8 (PMC5722738; doi:10.1007/s13280-017-0969-8)
Supplement: Supplementary file 1 — Supplementary material 1 (PDF 194 kb) [file 13280_2017_969_MOESM1_ESM.pdf]

Ambio

Electronic Supplementary Material

Title: Lower dietary phosphorus supply in pigs match both animal welfare aspects and resource efficiency

Authors: Michael Oster, Christian Gerlinger, Kaja Heide, Franziska Just, Luisa Borgelt, Petra Wolf, Christian Polley, Brigitte Vollmar, Eduard Muráni, Siriluck Ponsuksili, Klaus Wimmers

Table S1 Analysed composition of the experimental diets.

Item	Unit	Low	Medium	High
Dry matter	%	89.8	89.7	90.5
Crude protein	% of DM	19.6	19.6	19
Crude ash	% of DM	4.97	6.15	7.15
Calcium	% of DM	0.79	1.27	1.69
Phosphorus	% of DM	0.56	0.84	1.02
Digestible phosphorus	% of DM	0.31	0.49	0.6
Calcium: phosphorus ratio		1.41	1.51	1.65
Phytase activity	IU/kg	253	251	253
Metabolisable energy	ME: MJ/kg	14.7	14.6	14.3

Table S2 Primer used for qPCR

Gene symbol	Sequence 5' - 3' For	Sequence 5' - 3' Rev	size (bp)
VDR	ACCTGGATCTGAGCGAAGAA	GATGGCACTTGACTTCAGCA	194
Cyp24A1	GCTGGACAACAAAATCAATGAG	CTCATAGAGCACAAGGCAGATG	142
Cyp27A1	GGCCCTGTACCATCTTTCAA	TTGTCTGTGATGACCCTGGA	192
Cyp27B1	GCATCTCTCCCCTTTGGCTT	GGGTCATGGGTCTGATTGGG	145
PTH1R	GGCGTCCATTACATCGTCTT	AAAGTCCAGTGCCAATGTCC	198
THRA	TGGAAAGCGAAAAAGAAAGAAC	TGAGTAGGTGGGATGGAGATTC	202
SLC34A1	TGGGCTTGTGTGACTGAGAG	CCCAGTCAGAGTTGTGCGTA	198
SLC34A2	GAATCAGCCCGAAACAAGAG	AAACCATCCGTCCAACAGAG	128
SLC34A3	TCGTCCTGGTCACAGTCC	CGGGGTTCTCATAGCAGTG	192
